# Analysis of correlative factors of female coronary slow-flow phenomenon: A retrospective study

**DOI:** 10.1097/MD.0000000000038262

**Published:** 2024-05-24

**Authors:** Xin Chen, Alian Zhang, Zuojun Xu, Zhaofang Yin, Changqian Wang, Junfeng Zhang, Ling Bian

**Affiliations:** a Department of Cardiology, Shanghai Ninth People’s Hospital, Shanghai JiaoTong University School of Medicine, Shanghai, China.

**Keywords:** apolipoprotein E (ApoE), coronary slow-flow phenomenon, female, free fatty acid, free thyroxine (FT4)

## Abstract

The coronary slow-flow phenomenon (CSFP) is a manifestation of coronary artery disease wherein coronary angiography reveals no apparent stenosis; however, there is a delay in blood flow perfusion. Given its increased occurrence in male patients, with the majority of subjects in previous studies being male, this study aimed to explore whether distinct risk factors are present in female patients with CSFP. This single-center retrospective study focused on female patients diagnosed with CSFP by using coronary angiography. Eligible patients meeting the predefined inclusion and exclusion criteria were divided into the study group (presenting with CSFP) and control group (displaying normal epicardial coronary arteries). Comparative analyses of clinical and diagnostic data were performed. Ninety-two patients with CSFP and an equal number of controls were enrolled in this study. Patients with CSFP exhibited a higher prevalence of smokers (*P* = .017) and a heightened incidence of diabetes mellitus (DM) (*P* = .007). Significantly elevated levels of total cholesterol (TC) (*P* = .034) and free fatty acids (FFA) (*P* = .016) were observed in the CSFP group compared to those in the control group. Additionally, patients with CSFP displayed lower levels of apolipoprotein E (ApoE) (*P* = .092), free thyroxine (FT4) (*P* = .001), and total thyroxine (TT4) (*P* = .025). Logistic regression analysis indicated that smoking (*P* = .019), FFA (*P* < .001), ApoE (*P* = .015), and FT4 (*P* < .001) were independent risk factors for CSFP, accounting for confounding factors. Additionally, the area under the ROC curve (AUC) of the combined effect of smoking, ApoE, FT4, and FFA on CSFP was 0.793 (95% CI: 0.729–0.857, *P* < .01). In addition to the established risk factors for smoking, diabetes, and hyperlipidemia, female patients with CSFP exhibited significant differences in apoE, FFA, FT4, and TT4 levels compared to the control group. Smoking, FFA, and FT4 levels emerged as independent risk factors for CSFP.

## 1. Introduction

The coronary slow-flow phenomenon (CSFP) is an angiographic manifestation characterized by the gradual passage of contrast in the absence of obstructive coronary artery disease, initially proposed by Tambe et al.^[1]^ Despite its recognition, its underlying pathogenesis remains unclear. The occurrence and progression of CSFP may involve coronary microcirculation disorders, endothelial function impairment, inflammation, oxidative stress, atherosclerosis, and an imbalance in vasoactive substances.^[2,3]^ In coronary arteriography, CSFP is observed in 1% to 7% of patients with chest pain, and 4% of those with unstable angina also exhibit CSFP.^[4–6]^ Individuals affected by CSFP experience recurrent chest pain, frequent hospitalizations, and repeated cardiac catheterizations.^[7]^

Previous investigations have identified various risk factors for CSFP, including sex, smoking, age, diabetes, hypertension, inflammation, and hyperlipidemia. Notably, CSFP is more prevalent in male patients, with twice as many males as females affected.^[2]^ Consequently, most of the research participants were male, leaving a limited representation of females. Theoretically, females may exhibit distinct characteristics. A retrospective analysis of the clinical data was performed to explore the features of female patients with CSFP.

## 2. Methods

### 2.1. Study design and patients

This was a single-center retrospective observational study. The study sample was retrospectively selected from Shanghai JiaoTong University School of Medicine, Shanghai Ninth People’s Hospital, covering the period from January 2016 to September 2022, with a specific focus on female patients.

Female patients with angiography indicating vessel stenosis of <40% and demonstrating CSFP in at least one vessel were included in the CSFP group. Female patients who had chest tightness or chest pain, or for other reasons requiring identification of coronary artery status and were confirmed with normal epicardial coronary arteries were enrolled in the control group. The exclusion criteria were as follows: ST-segment elevation myocardial infarction and non-ST-segment elevation myocardial infarction; previously established coronary heart disease; malignant tumors; acute or chronic infection or inflammation; declined renal function, estimated glomerular filtration rate (eGFR) ≤ 30 mL/minutes, or undergoing hemodialysis; severe hepatic dysfunction (glutamic-pyruvic transaminase 3 times higher); and thyroid disorders.

The study protocol was approved by the Ethics Committee (No. SH9H-2021-T1-1), Shanghai Jiao Tong University School of Medicine, and adhered to the principles of the Declaration of Helsinki. Written informed consent was obtained from all patients.

### 2.2. Data collection and definition

Blood samples were obtained in the morning after overnight fasting during hospitalization, encompassing routine blood counts, liver and kidney function, glucose glycated hemoglobin, lipid profiles, and thyroid function. eGFR was calculated using the CKDEPI equation.

### 2.3. Angiogram and analysis

Coronary angiography followed established procedures, with quantitative coronary angiography^[8]^ performed by 2 independent physicians using the Cardiovascular Angiography Analysis System 5.10. It should be noted that, in patients with CSFP on the first scan, we administered nitrate and repeat imaging, then quantitative coronary angiography. The minimal luminal diameter was defined as the smallest lumen diameter in the lesion segment, whereas the reference vessel diameter was defined as the average diameter of the proximal and distal coronary segments without evident narrowing.

Coronary blood flow was quantitatively assessed using the corrected TIMI frame count (cTFC). The criteria for CSFP were met when coronary angiography showed a normal or narrowed coronary artery ≤ 40%. For an image acquisition speed of 30 frames/s, CSFP diagnosis was established if the cTFC exceeded 27 frames in at least one artery. Interpretation of the coronary angiography images was conducted by 2 experienced cardiologists.

### 2.4. Statistical analysis

All statistical analyses were conducted using SPSS26 (SPSS, IBM, Armonk, NY). Quantitative variables are presented as mean ± SD for normally distributed data, whereas nonnormally distributed data are described as medians and quartiles. Qualitative variables are expressed as numbers and percentages unless otherwise specified.

For the analysis of quantitative variables, an unpaired 2-tailed Student *t* test or Mann–Whitney *U* test was employed based on data distribution characteristics, whether normal or not. A 2-sided chi-square test was used to compare the qualitative variables. Univariate and multivariate logistic regression analyses were performed to identify independent predictors of CSFP. Variables with *P* < .1 in the univariate analysis were selected for multivariable analysis, and the results were expressed as odds ratios (ORs) with corresponding 95% confidence intervals (CI).

Receiver Operating Characteristic (ROC) curves were employed to illustrate the sensitivity and specificity, along with optimal cutoff points for predicting CSFP. All statistical tests were conducted at a significance level of *P* < .05.

## 3. Results

### 3.1. Baseline clinical characteristics

**Figure 1.**
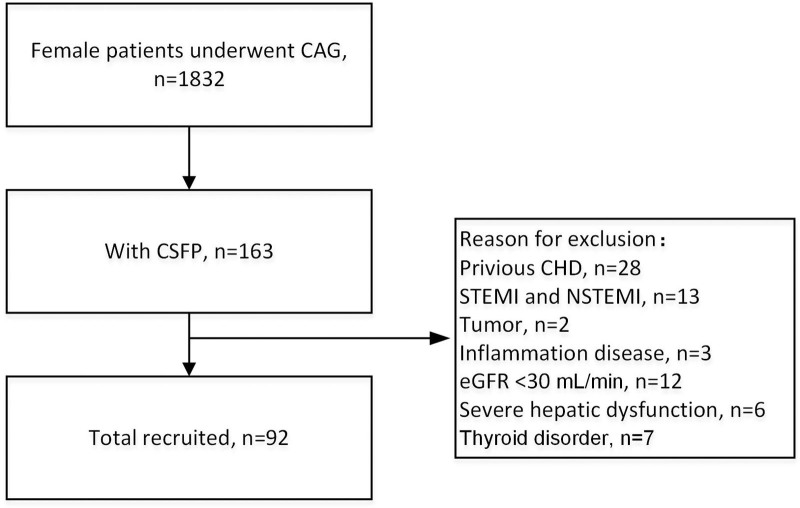
Study flowchart. CAG = coronary arteriography, CHD = coronary heart disease, CSFP = coronary slow-flow phenomenon, eGFR = glomerular filtration rate, NSTEMI = non-ST-segment elevation myocardial infarction, STEMI = ST-segment elevation myocardial infarction.

A total of 1832 female patients underwent coronary angiography, 163 of whom exhibited CSFP. Following the application of the exclusion criteria, 71 patients were excluded, resulting in a final study sample of 92 female patients with CSFP (Fig. 1). The control group was comprised of 92 patients with normal epicardial coronary arteries.

Compared to the control group, the CSFP group had a higher prevalence of smokers (32.6% vs 17.4%, *P* = .017) and a higher incidence of diabetes mellitus (DM) (21.7% vs 7.6%, *P* = .007). No significant differences were observed in age, menopausal status, hypertension, hyperlipidemia, body mass index, left ventricular ejection fraction, systolic blood pressure, or diastolic blood pressure between the CSFP and control groups. In the CSFP group, CSFP was observed in 70.7% of the cases in the left anterior descending artery (LAD), 46.7% in the right coronary artery (RCA), and 22.8% in the left circumflex artery. The baseline clinical characteristics of the patients are shown in Table 1.

**Table 1:** Baseline characteristics of patients.

	Control (n = 92)	CSFP (n = 92)	*P* value
Age, yr	60.37 ± 14.48	60.72 ± 9.31	.839
Menopause	73 (79.3%)	76 (82.6%)	.573
Smoking, n (%)	16 (17.4%)	30 (32.6%)	.017
CHD family history, n (%)	3 (3.3%)	8 (8.7%)	.120
Hypertension, n (%)	42 (45.7%)	50 (54.3%)	.461
DM, n (%)	7 (7.6%)	20 (21.7%)	.007
Hyperlipidemia, n (%)	12 (13.0%)	8 (8.7%)	.851
SBP, mm Hg	134.01 ± 18.89	131.58 ± 18.71	.381
DBP, mm Hg	80.57 ± 13.06	80.29 ± 12.40	.885
LVEF (%)	61.24 ± 3.28	61.28 ± 3.47	.941
BMI	24.83 ± 5.25	25.41 ± 4.31	.424
Vessel distribution			
LAD, n (%)		65 (70.7%)	
LCX, n (%)		21 (22.8%)	
RCA, n (%)		43 (46.7%)	
TIMI frame count			
LAD (corrected)	15.64 ± 4.79	34.28 ± 10.87	<.001
RCA	14.33 ± 3.64	32.30 ± 7.73	<.001
LCX	16.00 ± 5.01	33.48 ± 9.6	<.001

Values were represented by mean ± SD, or n (%).

BMI = body mass index, CHD = coronary heart disease, DBP = diastolic blood pressure, DM = diabetes mellitus, LAD = left anterior descending branch, LCX = left circumflex artery, LVEF = left ventricular ejection fraction, RCA = right coronary artery, SBP = systolic blood pressure.

Significant disparities in laboratory findings, including total cholesterol (TC), free fatty acids (FFA), apolipoprotein E (ApoE), free thyroxine (FT4), and total thyroxine (TT4) levels, were observed between the CSFP and control groups. The CSFP group exhibited elevated TC (4.21 ± 0.93 vs 3.92 ± 0.93, *P* = .034) and FFA (0.62 ± 0.18 vs 0.48 ± 0.18, *P* = .016) levels, while ApoE (3.99 ± 1.76 vs 4.40 ± 1.51, *P* = .092), FT4 (0.89 ± 0.16 vs 0.95 ± 0.18, *P* = .001), and TT4 (7.97 ± 1.40 vs 8.46 ± 1.56, *P* = .025) levels were significantly lower compared to the control group. No significant differences were observed in other laboratory findings such as white blood cell count, C-reactive protein (CRP), brain natriuretic peptide, glutamic-pyruvic transaminase, albumin, eGFR, low-density lipoprotein cholesterol, high-density lipoprotein cholesterol, triglycerides, apolipoprotein A-I, apolipoprotein B, and thyroid-stimulating hormone (TSH) (Table 2).

**Table 2:** Laboratory findings among CSFP and control groups.

	Control (n = 92)	CSFP (n = 92)	*P* value
WBC	6.28 ± 1.54	6.49 ± 1.75	.392
C-reactive protein	2.57 ± 2.49	2.77 ± 2.90	.617
Hemoglobin	143.66 ± 15.23	146.96 ± 15.13	.153
Blood platelet	205.44 ± 46.81	202.90 ± 42.26	.706
BNP	67.57 ± 87.28	59.68 ± 99.34	.568
FBG	5.58 ± 1.54	5.24 ± 0.93	.078
HbA1c	6.10 ± 0.94	6.08 ± 0.80	.859
GPT	26.51 ± 24.40	25.32 ± 15.41	.692
Albumin	40.36 ± 3.41	40.20 ± 3.43	.747
eGFR	81.79 ± 17.15	77.48 ± 20.89	.127
Uric Acid	361.45 ± 102.81	375.20 ± 96.51	.351
Urea nitrogen	5.81 ± 1.83	5.78 ± 1.80	.926
TC, mmol/L	3.92 ± 0.93	4.21 ± 0.93	.034
LDL-C, mmol/L	2.69 ± 0.77	2.79 ± 0.91	.435
HDL-C, mmol/L	1.04 ± 0.31	0.98 ± 0.26	.164
TG, mmol/L	1.58 ± 0.83	1.74 ± 1.20	.312
Lipoprotein a	0.17 ± 0.55	0.14 ± 0.14	.703
Apolipoprotein A-I	1.11 ± 0.23	1.09 ± 0.18	.486
Apolipoprotein B	0.78 ± 0.20	0.82 ± 0.22	.174
ApoE	4.40 ± 1.51	3.99 ± 1.76	.092
FFA	0.48 ± 0.18	0.62 ± 0.18	.016
TSH	1.91 ± 1.87	2.07 ± 1.67	.548
FT3	3.12 ± 0.35	3.13 ± 0.36	.929
FT4	0.95 ± 0.18	0.88 ± 0.16	.002
TT3	0.90 ± 0.15	0.91 ± 0.18	.721
TT4	8.46 ± 1.56	7.97 ± 1.40	.025

Values were represented by mean ± SD, or n (%).

ApoE = apolipoprotein E, BNP = Brain natriuretic peptide, eGFR = estimated glomerular filtration rate, FBG = fasting blood-glucose, FFA = free fatty acid, FT3 = free triiodothyroxine, FT4 = free thyroxine, GPT = glutamic-pyruvic transaminase, HbA1c = glycated hemoglobin, HDL-C = high-density lipoprotein cholesterol, LDL-C = low-density lipoproteins-cholesterol, TC = total cholesterol, TG = triglycerides, TSH = thyroid-stimulating hormone, TT3 = total triiodothyroxine, TT4 = total thyroxine, WBC = white blood cell.

### 3.2. Multivariate logistic regression analysis

Univariate and multivariate logistic regression analyses were conducted to ascertain the potential influencing risk factors in patients with CSFP as shown in Table 3. In univariate analysis, smoking, diabetes, total cholesterol, ApoE, FFA, FT4, and TT4 were significantly associated with CSFP. Furthermore, the multivariable logistic regression model revealed that smoking status (OR: 2.672, 95% CI: 1.178–6.061, *P* = .019), FFA (OR: 343.243, 95% CI: 30.423–3872.591, *P* < .001), ApoE (OR: 0.758, 95% CI: 0.607–0.948, *P* = .015), and FT4 (OR, 0.034; 95% CI: 0.002–0.520, *P* = .015) were independent risk factors for CSFP after adjusting for confounding factors.

**Table 3:** Univariate and multivariate logistic regression model for prediction of CSFP.

Variable	Univariate analysis OR (95% CI)	*P* value	Multivariate analysis OR (95% CI)	*P* value
Smoking	2.298 (1.149–4.597)	.019	2.672 (1.178–6.061)	.019
DM	3.373 (1.349–8.432)	.009	2.750 (0.957–7.899)	.060
Total cholesterol	1.411 (1.023–1.946)	.036	1.383 (0.904–2.117)	.135
ApoE	0.852 (0.705–1.030)	.098	0.758 (0.607–0.948)	.015
FFA	111.797 (15.975–782.400)	<.001	343.243 (30.423–3872.591)	<.001
FT4	0.057 (0.009–0.384)	.003	0.034 (0.002–0.520)	.015
TT4	0.795 (0.649–0.975)	.028	0.760 (0.564–1.023)	.070

ApoE = apolipoprotein E, DM = diabetes mellitus, FFA = free fatty acid, FT4 = free thyroxine, TC = total cholesterol, TT4 = total thyroxine.

### 3.3. ROC curve analysis for CSFP

**Figure 2.**
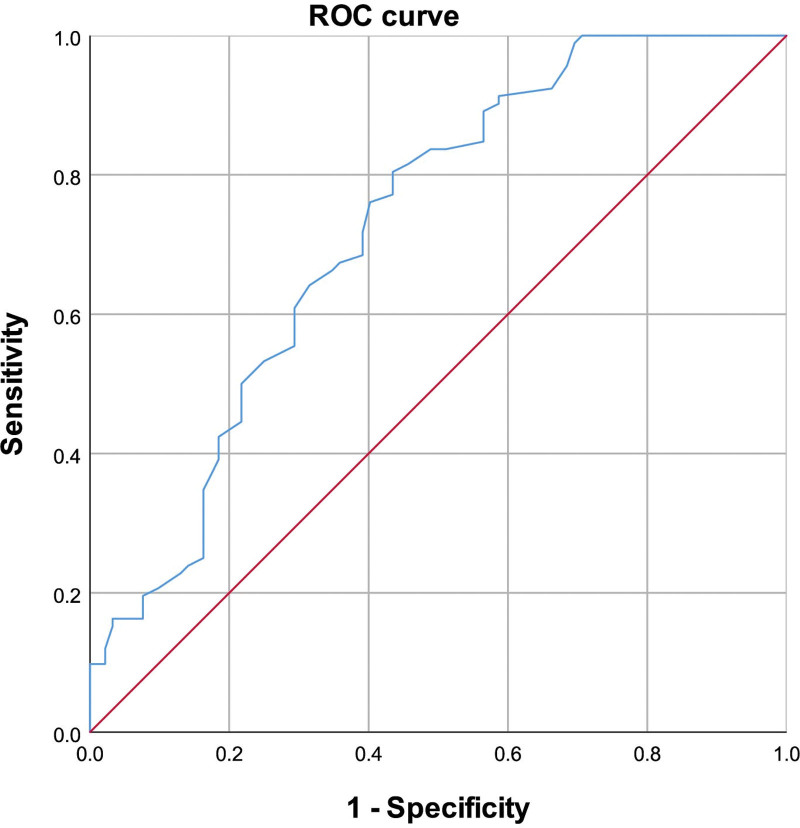
Receiver operating characteristic curve of FFA for CSFP. The area under the ROC curve (AUC) was 0.723 (95% CI: 0.650–0.796, *P* < .01). CSFP = coronary slow-flow phenomenon, FFA = free fatty acid.

**Figure 3.**
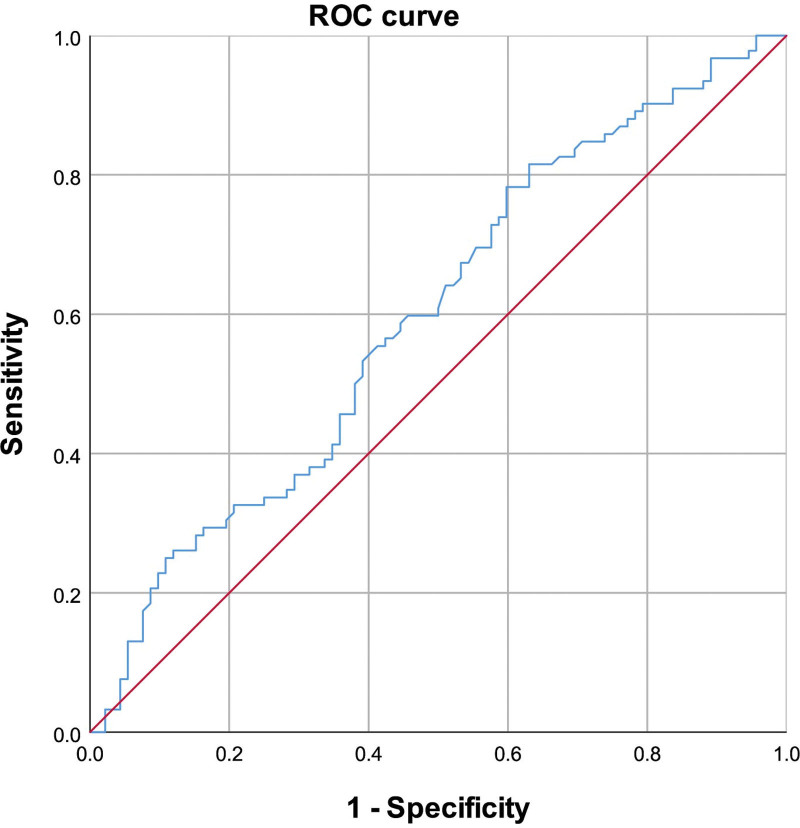
Receiver operating characteristic curve of ApoE for CSFP. The area under the ROC curve (AUC) was 0.596 (95% CI: 0.515–0.678, *P* = .24). ApoE = apolipoprotein E, CSFP = coronary slow-flow phenomenon.

**Figure 4.**
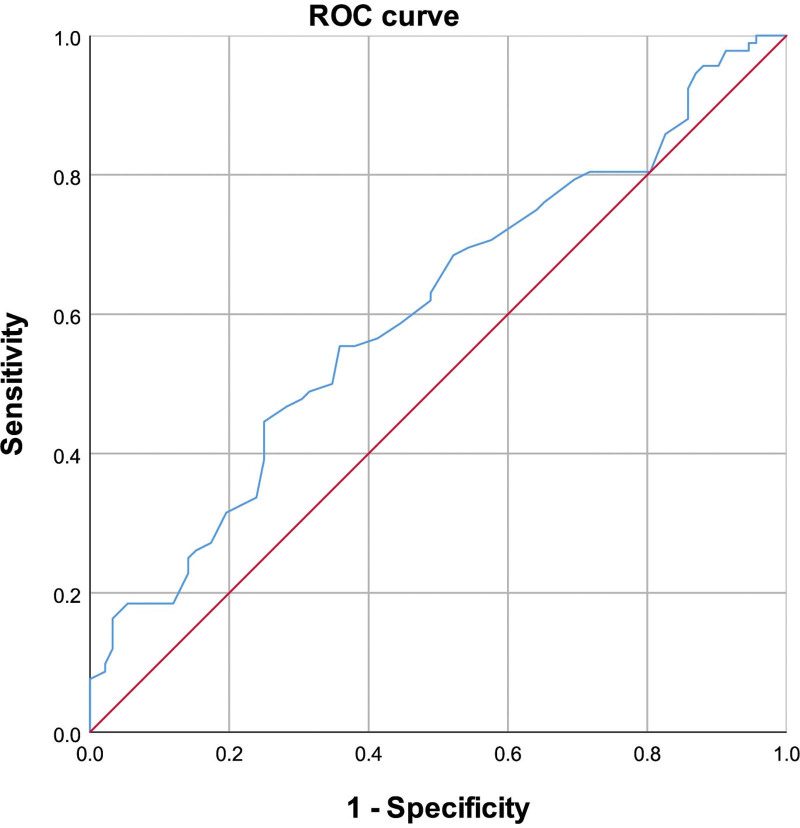
Receiver operating characteristic curve of FT4 for CSFP. The area under the ROC curve (AUC) was 0.604 (95% CI: 0.523–0.686, *P* < .01). CSFP = coronary slow-flow phenomenon, FT4 = free thyroxine.

**Figure 5.**
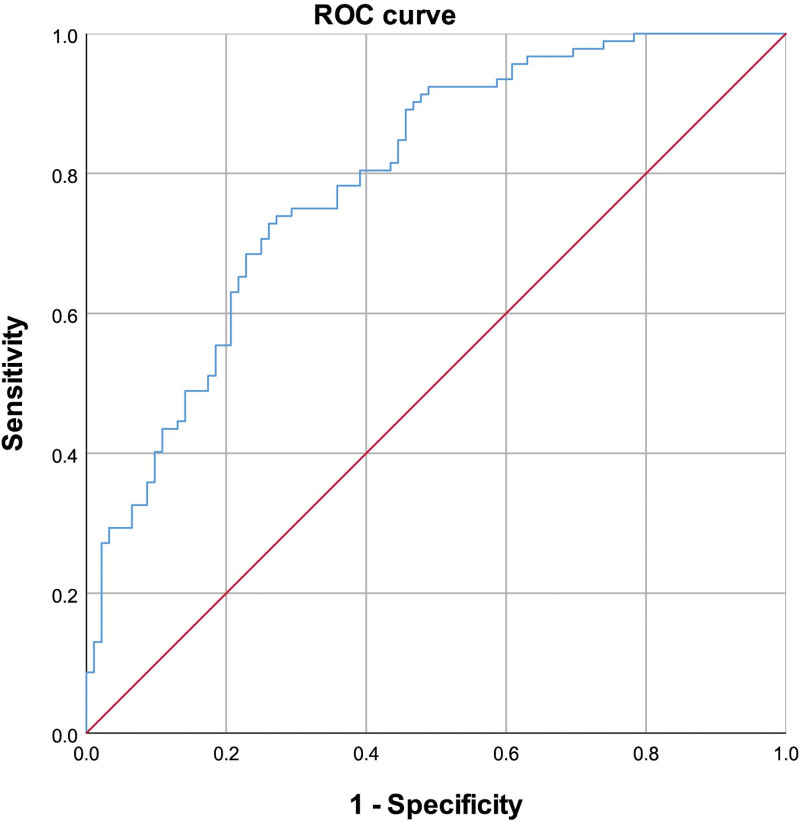
Receiver operating characteristic curve of combined effect of smoking, ApoE, FT4 and FFA for CSFP. The Area Under the ROC Curve (AUC) of the combined effect was 0.793 (95% CI: 0.729–0.857, *P* < .01). ApoE = apolipoprotein E, CSFP = coronary slow flow phenomenon, FFA = free fatty acid, FT4 = free thyroxine.

ROC analysis was conducted to assess the sensitivity and specificity of ApoE, FFA, and FT4 levels for predicting CSFP. The area under the ROC curve (AUC) for FFA was 0.723 (95% CI: 0.650–0.796, *P* < .01) (Fig. 2); For ApoE, the AUC was 0.596 (95% CI: 0.515–0.678, *P* = .24) (Fig. 3) and 0.604 (95% CI: 0.523–0.686, *P* < .01) for FT4 (Fig. 4). Additionally, the combined effect of smoking, ApoE, FT4, and FFA on CSFP was analyzed, revealing an AUC of 0.793 (95% CI: 0.729–0.857, *P* < .01) (Fig. 5). The optimal cutoff demonstrated 80.4% sensitivity and 70.9% specificity.

## 4. Discussion

CSFP is diagnosed through the observation of slow contrast material flow during angiography in coronary arteries that are either normal or nearly normal.^[1]^ Although there was no significant difference in left ventricular systolic function between patients with CSFP and those with normal coronary artery, patients with SCFP can present with various conditions such as unstable angina, acute myocardial infarction, and ventricular tachycardia.^[9–11]^ Although CSFP is more prevalent in men, it is not exclusive to men, as it can also manifest in women. This study exclusively enrolled female patients to investigate the specific characteristics of CSFP.

We systematically assessed and compared the baseline characteristics of female patients with CSFP with those in the control group. Our findings revealed a higher prevalence of smokers and an increased incidence of DM in female patients.^[12]^ The distribution pattern of CSFP across the LAD, circumflex, and RCA aligns with previous studies, with the LAD being the most affected vessel and the RCA the second most affected vessel.^[13]^ Significant differences in TC, ApoE, FFA, FT4, and TT4 levels were observed between the CSFP and control groups. Subsequently, we aimed to establish independent associations between smoking, apoE, FFA, and FT4 levels and CSFP. Smoking and FFA were identified as risk factors, whereas ApoE and FT3 emerged as protective factors for CSFP, independent of various conventional risk factors.

As an early manifestation of atherosclerosis, it is often associated with multiple cardiovascular risk factors.^[14]^ Diabetes and smoking were identified as independent risk factors for cardiovascular disease, whereas disorders in lipid metabolism independently predicted the risk of atherosclerotic cardiovascular disease.^[15]^ Our study revealed a higher prevalence of smoking and an increased incidence of diabetes among female patients with CSFP, which is consistent with the findings in male patients and previous studies.

Dyslipidemia has long been recognized as a major risk factor for cardiovascular diseases, primarily because of its role in the pathophysiology of large- and medium-sized atherosclerosis.^[16]^ A growing scientific consensus suggests that dyslipidemia may lead to microvascular dysfunction before manifesting as overt atherosclerosis.^[17]^ In line with this, our study demonstrated abnormal lipid metabolism indices among patients with CSFP, including increased total cholesterol, decreased apoE, and significantly elevated plasma FFA concentrations (*P* < .01).

Inflammation is a risk factor for several cardiovascular diseases, and inflammatory responses have also been observed in CSFP. Plasma soluble adhesion molecules and inflammatory markers were significantly elevated in patients with CSFP, including CRP and interleukin-6, matrix metallopro-teinase-9, soluble CD40 ligand, and CRP to albumin ratio.^[18,19]^ However, in our study there was no significant difference in CRP between the 2 groups. This may be due to the unique characteristics of females. But further study is needed.

### 4.1. ApoE in CSFP patients

Compared with the control group, ApoE levels were significantly diminished in patients with CSFP. ApoE, which belongs to the class of apolipoproteins present in chylomicrons and intermediate-density lipoproteins, facilitates the transfer of triglyceride-rich lipoprotein components. Its crucial role extends beyond cholesterol transport and encompasses diverse antioxidant, anti-inflammatory, and immunomodulatory effects. These aspects are integral to the protective function of ApoE against inflammation and disease.^[20]^ Notably, this study is the first to reveal a correlation between ApoE levels and CSFP in female patients.

### 4.2. FFA in CSFP patients

FFA are closely associated with the onset and progression of cardiovascular disease. In a prospective cohort study involving 1221 elderly individuals in Sweden, FFA levels were significantly associated with the risk of cardiovascular disease-related mortality. Elevated serum FFA levels have been linked to mortality due to acute myocardial infarction.^[21]^ Similarly, a prospective cohort study conducted in China established a nonlinear U-shaped relationship between baseline FFA levels and mortality or ischemic events in coronary artery disease patients with type 2 DM.^[22]^ In our study, elevated FFA levels were identified in the CSFP group, indicating that it is an independent risk factor for CSFP. A comprehensive assessment of FFA and traditional risk factors may aid in the identification of high-risk individuals requiring close monitoring and proactive intervention.^[23]^

### 4.3. Hypothyroidism and FT4 in CSFP patients

Hypothyroidism, a systemic hypometabolic syndrome rooted in thyroid hormone or thyroid hormone resistance, is implicated in various causative factors.^[24]^ Both clinical and subclinical hypothyroidism are recognized as risk factors for atherosclerosis and cardiovascular diseases, contributing to an increased coronary artery disease (CAD) incidence and all-cause mortality.^[25]^ This association is attributed to the influence of thyroid hormones on lipid metabolism and alterations in vascular endothelial function.^[26]^ In our study, the CSFP group exhibited decreased FT4 levels compared with the control group. Endothelial injury within the coronary vasculature, which is the mechanism underlying CSFP, may induce coronary constriction and intravascular thrombosis. However, the precise mechanism through which hypothyroidism affects endothelial function remains unclear. Tian et al^[27]^ demonstrated that elevated TSH levels can induce endothelial cell dysfunction, potentially leading to atherosclerosis formation and development. The presence of thyrokinin receptors in human aortic smooth muscle cells suggests that thyroid hormones can directly influence the vascular bed, thereby affecting vasomotor function.^[28]^ Previous studies have also indicated that hypothyroidism is associated with a low level of systemic inflammatory response, serving as a potential trigger for endothelial dysfunction.^[29]^

### 4.4. Multivariate and ROC analyses for predictive factors in CSFP

Multivariate and ROC analyses were employed to explore the independent predictive roles of smoking, ApoE, FFA, and FT4 in CSFP. These findings indicate that FFA level is a robust independent predictor of CSFP. Additionally, we examined the combined effect of smoking and ApoE, FT4, and FFA levels on CSFP, revealing an area under the ROC curve of 0.793 (95% CI: 0.729–0.857, *P* < .01).

## 5. Limitations

This study has several limitations. First, it adopted a retrospective design with a relatively small sample size, thereby impeding generalizability. Second, owing to its observational nature, the establishment of precise causal relationships is unattainable. Third, the absence of short- and long-term patient follow-ups constitutes an additional limitation of this study. A prospective, large-scale investigation with comprehensive follow-up data analysis is imperative.

## 6. Conclusion

In conclusion, our study sought to delineate the clinical characteristics of female patients with CSFP. We identified smoking, FFA, apolipoprotein E (apoE), and FT4 as independent risk factors for CSFP. This knowledge enhances our understanding of CSFP in female patients and forms a foundation for future endeavors in its prevention and treatment.

## Author contributions

**Conceptualization:** Xin Chen, Changqian Wang, Ling Bian.

**Data curation:** Xin Chen, Alian Zhang, Changqian Wang.

**Formal analysis:** Xin Chen, Alian Zhang.

**Funding acquisition:** Alian Zhang.

**Investigation:** Xin Chen, Zuojun Xu, Zhaofang Yin.

**Methodology:** Xin Chen, Zuojun Xu, Zhaofang Yin, Changqian Wang.

**Project administration:** Xin Chen, Zuojun Xu.

**Resources:** Zuojun Xu, Zhaofang Yin, Junfeng Zhang.

**Supervision:** Zuojun Xu, Zhaofang Yin, Changqian Wang, Junfeng Zhang.

**Software:** Zhaofang Yin, Junfeng Zhang.

**Validation:** Zhaofang Yin, Junfeng Zhang.

**Visualization:** Xin Chen, Zhaofang Yin, Junfeng Zhang.

**Writing – original draft:** Xin Chen, Alian Zhang, Ling Bian.

**Writing – review & editing:** Changqian Wang, Ling Bian.
